# Dynamics Changes in Physicochemical Properties, Antioxidant Activity, and Non-Volatile Metabolites During Bulang Pickled Tea Fermentation

**DOI:** 10.3390/foods14050878

**Published:** 2025-03-04

**Authors:** Jinping Zhou, Laifeng Chen, Fan Zhang, Hooiling Foo, Zhenhui Cao, Qiuye Lin

**Affiliations:** 1 College of Food Science and Technology, Yunnan Agricultural University, Kunming 650201, China; zhou_jinping101@163.com (J.Z.); chenlaifeng2025@126.com (L.C.); 2 Tea Research Institute, Hunan Academy of Agricultural Sciences, Changsha 410125, China; zhangfan502@126.com; 3 Department of Bioprocess Technology, Faculty of Biotechnology & Biomolecular Sciences, Universiti Putra Malaysia, 43400 UPM Serdang, Selangor, Malaysia; hlfoo@upm.edu.my; 4 Research Laboratory of Probiotics and Cancer Therapeutics, UPM-MAKNA Cancer Research Laboratory (CANRES), Institute of Bioscience, Universiti Putra Malaysia, 43400 UPM Serdang, Selangor, Malaysia; 5 Faculty of Animal Science and Technology, Yunnan Agricultural University, Kunming 650201, China

**Keywords:** pickled tea, taste quality, physiochemical, antioxidant activity, metabolomics

## Abstract

The present study investigated the dynamics changes in physicochemical properties and non-volatile metabolites during Bulang pickled tea fermentation. A combination of artificial sensory evaluation, chemical-physical analysis, ultra performance liquid chromatography coupled with quadrupole/time-of-flight mass spectrometry (UPLC-QTOF-MS), and multivariate statistical analysis were employed to examine the differences among four fermentation stages of Bulang pickled tea. The bitterness, astringency, sweetness after taste, sourness and fermentation taste tended to increase with fermentation. The highest lactic acid bacteria, aerobic bacteria, total titratable acidity, total soluble sugar, total polyphenols, and total flavonoids were recorded at the second month of fermentation, while fungi, total free amino acids, total antioxidant capacity and hydroxyl free radical scavenging capacity increased with fermentation. Mantel test demonstrated significant associations between lactic acid bacteria /fungal communities and taste characteristics. UPLC-QTOF-MS analysis led to the identification of 35 differential non-volatile metabolites, predominantly comprising heterocyclic compounds, organic acids with their derivatives, and flavonoids. Nine non-volatile metabolites are related to antioxidant activity, and morin, malvidin and 7-methylxanthine exhibit relatively strong antioxidant activity. This study provides comprehensive insights into the non-volatile metabolites and antioxidant function of Bulang pickled tea.

## 1. Introduction

Yunnan in southwest China is the province with the greatest number of ethnic minorities in China and is known as well for its traditional fermented foods. Pickled tea, a traditional fermented food of the Bulang ethnic group in Yunnan, undergoes an-aerobic microbial fermentation of tea leaves (*Camellia sinensis*) [1]. The Bulang ethnic group has been deeply rooted in tea culture and the history of Bulang pickled tea (called mian) dates back more than 1700 years [2]. Grounded in comprehensive literature review and empirical investigations in ethnic areas of Bulang group, Bulang pickled tea is generally prepared as follows: after withering, steaming, and air-drying freshly picked tea leaves to reduce moisture content, the leaves are carefully packed in bamboo buckets, sealed, and buried underground for anaerobic fermentation for three months [3]. In contrast to other kinds of pickled tea, such as Chinese De’ang pickled tea, Myanmar *Laphet*, and Japanese *Awa-bancha*, Bulang pickled tea is produced without rolling prior to anaerobic fermentation and less cell deconstruction and release of chemical compounds may happen, which might consequently impact the product properties.

Fermentation alters the physiochemical properties and the quality of pickled tea. Polysaccharides degraded by microbial enzyme provides a carbon source for microorganism growth and organic acids production, as observed with pH decline and acidity increase during *Miang* fermentation [4]. The tea components including teapolyphenols, alkaloids, amino acids, and carbohydrates, are the chemical nature of the taste and color of tea infusion as well as texture of tea leaves [5]. The increment in components such as catechins and phenolic acids and the reduction in soluble sugars may bring a strong bitterness and astringency and weak sweet aftertaste in pickled tea [6,7]. The oxidation of catechins might be associated with decreased lightness of Miang tea leaves [8]. In our recent study, the microbial communities in Bulang pickled tea, especially *Lactobacillus plantarum* and *Cladosporium sphaerospermum*, may shape its vola-tomic volatomic profile and contribute the aroma development, highlighting that the chemical changes during anaerobic fermentation impact the taste of Bulang pickled tea [3]. Therefore, it is imperative to investigate the interaction of the taste characteristics and chemical composition of Bulang pickled tea to facilitate an understanding of the mechanism underlying its flavor quality formation. 

In addition, the non-volatile metabolites produced during fermentation of pickled tea may lead to improvement in the bioactivity. A study with four types of post-fermented tea prepared under aerobic, anaerobic or combination has shown that anaerobic fermentation may benefit the antioxidant activity of tea due to the production of novel antioxidant metabolites such as resorcinol [9]. It is worth noting that an-aerobic fermentation facilitates the accumulation of a wide range of compounds in pickled tea including amino acids, organic acids, flavonoids, flavonoid glycosides, geranium glycosides and geranium glycosides, as well as epigallocatechin [10]. Then, among these enriched components in pickled tea, glycosides, organic acids, catechins, and flavonoids are associated with the α-amylase and α-glucosidase suppressing bioactivities [11]. The above findings indicate that study on non-volatile metabolites of pickled tea attributed its bioactivities would expand our understanding on effect of anaerobic fermentation modulating the potential health benefits of tea products. 

It is well documented that the composition and contents of metabolites during fermentation have a significant impact on both taste formation and antioxidant activity of tea products [12]. We speculate that chemical and non-volatile changes occur-ring during fermentation of Bulang pickled tea may affect its taste properties and antioxidant activity. Therefore, the present study aimed to explore the dynamic changes in the taste attributes and physiochemical measurements, antioxidant activity and metabolites with UHPLC-QTOF-MS during the fermentation process of Bulang pickled tea and identify the chemical basis of its taste quality and bioactivity. This study would provide a solid perspective on the mechanism underlying the sensory quality and activity function of Bulang pickled tea and hold substantial implications for advancing the pickled tea industry.

## 2. Materials and Methods

### 2.1. Chemicals and Reagents

Ascorbic acid, Folin-Ciocâlteu reagent, coomassie brilliant blue G-250, and five standard reagents gallic acid, (+)-catechin(C), D (+)-glucose, bovine serum albumin, L-glutamic acid, ABTS free radical scavenging capacity assay kit, DPPH free radical scavenging capacity assay kit, micro hydroxyl free radical scavenging capacity assay kit, and total antioxidant capacity (T-AOC) assay kit (FRAP method) were provided by Beijing Solarbio Science & Technology Co., Ltd. (Beijing, China). Anthrone and ninhy-drin were purchased from Shanghai Macklin Biochemical Technology Co., Ltd. (Shanghai, China). Methanol, formic acid, and acetonitrile, used for MS analysis and of chromatographic grade, were purchased from Merck (Darmstadt, Germany), while formic acid was bought from Thermo Fisher (Waltham, MA, USA). All remaining rea-gents were of analytical grade and sourced from China.

### 2.2. Tea Processing and Sample Collection

Samples of Bulang pickled tea were obtained from Banzhang Village of Bulangshan Township, Menghai County, Xishuangbanna Autonomous Prefecture, Yunnan Province in 2021. The fresh tea leaves with one bud and two leaves were picked and withered, followed by boiling for 20 min. After being cooling cooled down to ambient temperature and drained in a dry and well-ventilated environment, the tea leaves were placed into a bamboo tube, and sealed tubes followed fermentation underneath the ground. Tea samples were collected at 0, 1, 2, and 3 months of fermentation, then sun-dried for sensory evaluation. Bulang pickled tea fermented for 0, 1, 2, and 3 month (s) were collected and frozen in liquid nitrogen for 3 hours followed by storage at −80 °C prior to use. The samples for microbial counts were kept on the ice and transported to the laboratory. Tea samples collected in July, August, September, and October in 2021 were labeled as unfermented tea (B0), tea fermented for one month (B1), two months (B2), and three months (B3), respectively.

### 2.3. Taste Evaluation

Taste parameters of Bulang pickled tea was determined according to the Chinese National Standard (GB/T23776-2018) [13] by the panel consisting of tea evaluation experts (four males, six females, and aged between 23 and 50 years). Three grams of sun-dried Bulang pickled tea leaves were brewed with 150 mL of boiling pure water for 5 min. The sensory characteristics included five taste attributes bitterness, astringency, sweetness aftertaste, sourness, and fermentation taste. The taste of the tea samples was evaluated using a 9-point scale: 9, extremely strong; 7–8, strong; 5–6, neutral; 3–4, weak; 1–2, very weak; and 0, none [14]. The average score of each sensory attribute was used to create a radar diagram.

### 2.4. Enumeration of Viable Microbes

Twenty five grams of fresh tea sample and 225 mL of sterile tryptone-NaCl solution (0.1% *w/v* tryptone, 0.85% *w/v* NaCl) were homogenized in a TENLIN-D homogenizer (Jiangsu tenlin instrument Co., Ltd., Yancheng, China). Serial decimal dilution was performed and 100 μL of dilutions were spread onto the surface of different selective media. To count the number of total fungi, potato dextrose agar was incubated at 28 °C for 72 h, followed by a microscopic examination. Dilutions were plated onto Luria–Bertani agar and maintained at 28 °C for 48 h to enumerate total aerobic bacteria. Regarding counting of lactic acid bacteria (LAB), de Man, Rogosa, and Sharpe agar inoculated with dilutions were incubated at 37 °C for 48 h in an anaerobic jar (AnaeroJar TM 2.5L, Oxoid Ltd., Basingstoke, UK), including an anaeroPack (Mitsubishi Gas Chemical Co., Tokyo, Japan).

### 2.5. Determination of pH and Total Titratable Acidity

The pH value of Bulang pickled tea samples was determined using a Mettler Toledo PE28 pH meter (Mettler Toledo Technology (China) Co., Ltd., Shanghai, China), and the total titratable acidity (TTA) was assessed with a titration method. In brief, 5 g of the tea sample was suspended in 45 mL of sterile distilled water, homogenized for 10 min, and then, the supernatant was harvested through centrifugation at 10,000× *g* at 4 °C for 10 min. The supernatant was collected for pH measurement, and the total titratable acidity was determined through titration to neutral pH (pH 7.0) with 0.01 M NaOH, expressed as lactic acid equivalent.

### 2.6. Analysis of Chemical Composition

#### 2.6.1. Determination of Total Soluble Sugar

The total soluble sugar concentration in tea samples was measured based on the anthrone-sulfuric acid colorimetric method. The tea sample (1 g) was placed in 30 mL of boiling distilled water and soaked in a boiling water bath for 30 min. The tea infusion was filtered under reduced pressure while still hot, followed by cooling, and mixed with distilled water up to 50 mL. Eight milliliters of anthrone reagent (0.6 g anthrone, 100 mL sulfuric acid, and 33 mL distilled water) was added to 1 mL of the filtrate, and the mixture was kept at 100 °C for 7 min and then cooled down until room temperature. The absorbance was measured at 620 nm, and the content of total soluble sugar was calculated according to the standard glucose curve (μg/mL) and expressed as mg/g dry weight (dw).

#### 2.6.2. Determination of Soluble Protein

The soluble protein concentration of tea samples was determined based on the Coomassie brilliant blue G250 method. One gram of the sample was mixed with 80 mL of boiling distilled water and then subjected to extraction in a boiling water bath for 30 min. The tea infusion was filtered under reduced pressure while still hot, followed by cooling and mixed with distilled water up to 100 mL. Subsequently, 1 mL of the filtrate was combined with 5 mL of G250 solution (0.01%), and the absorbance was measured at 595 nm using a UV–visible spectrophotometer (UV-5100, Shanghai, China). Concentrations were determined based on a standard bovine serum albumin curve (μg/mL) and expressed as mg/g dry weight (dw).

#### 2.6.3. Determination of Total Free Amino Acids

The content of free amino acids of tea samples was evaluated using nihydrin colorimetry. Briefly, 5 mL of tea infusion prepared as described in Section 2.6.1 was placed in 2.5 mL of phosphate-buffered solution (pH 8.0) and 2.5 mL of 2% ninhydrin solution containing 0.8 mg/mL of tin chloride in a 25 mL volumetric flask and brought to volume. The optical density of the solution was measured at 570 nm. The concentration of total free amino acids in the sample was extrapolated from a standard L-glutamic acid curve (μg/mL) and expressed as mg/g dry weight (dw).

#### 2.6.4. Determination of Total Phenolics and Total Flavonoids

The extraction of total phenolics and total flavonoids was described by Liu, et al. [15] with simple modification. Five hundred milligrams of the sample was placed in 15 mL of a 60% (*v*/*v*) ethanol aqueous solution and extracted at 4 °C for 24 h. After centrifugation at 3000× *g* for 15 min, the supernatant was harvested and diluted with a 60% ethanol solution before measurement. All measurements were subsequently adjusted based on the dilution factor.

The concentration of total phenolics in tea extract was measured based on Folin–Ciocalteu method with gallic acid as the standard and expressed as mg/g dry weight (dw). Briefly, 0.5 mL of diluted tea extract was mixed with Folin–Ciocalteu reagent (diluted tenfold, 2.5 mL) and Na_2_CO_3_ solution (7.5%, 2 mL). After incubating in darkness for an hour, the absorbance at 765 nm was measured to ascertain the total phenolic content.

The determination of total flavonoids in tea extract was conducted using an AlCl_3_ colorimetric assay. Five hundred microliters of diluted tea extract was mixed with distilled water (3.5 mL) and 5% NaNO_2_ solution (150 μL), followed by shaking. Subsequently, 150 μL of 10% AlCl_3_ was added and allowed to react for 6 min before introducing 1 mL of 4% NaOH. The mixture was then maintained at ambient temperature for 15 min, and the optical density was measured at a wavelength of 510 nm. A standard curve was constructed using catechin and expressed as mg/g dry weight (dw).

### 2.7. Determination of Antioxidant Activities

Scavenging capacities of ABTS-free radicals, DPPH-free radicals, hydroxyl-free radicals, and T-AOC were determined according to the manufacturer’s instructions using commercial kits (Beijing Solarbio Science & Technology Co., Ltd., Beijing, China). Vitamin C (VC) solution served as the positive control for the determination of ABTS and DPPH scavenging activities. Half maximal inhibitory concentrations (IC50) of scavenging activities were analyzed to evaluate the antioxidant activities of ABTS-free radicals, DPPH-free radicals, and OH-free radicals. The T-AOC of the samples was calculated by sample mass (mmol/g). T-AOC was calculated using the following equation: T-AOC (mmol/g) = 34 (ΔA-0.1623)/4.6575/1000 W. ΔA represents sample absorbance minus blank absorbance.

### 2.8. Non-Targeted Metabolomics Analysis by UPLC-QTOF-MS

#### 2.8.1. Sample Preparation and Treatment

Four hundred microliters of 70% methanol solution in water containing the internal standard (L-2-chlorophenylalanine [2H3]-L-carnitine HCl, 4-fluoro-L-α-phenylglycine, L-phenylalanine (2-13C, 99%), [2H5]-hippuric acid, [2H5]-kynurenic acid, and [2H5]-phenoxy acetic acid) was mixed with 20 mg of the sample and vortexed for 3 min. The sample was sonicated in an ice bath for 10 min, followed by vortexing for 1 min, and then left at −20 °C for 30 min. Subsequently, the sample was centrifuged at 12,000 rpm for 10 min at 4 °C, and the pellet was removed. The supernatant was then centrifuged at 12,000 rpm for 3 min at 4 °C. Two thousand microliter supernatant was collected for LC–MS analysis.

#### 2.8.2. UPLC Conditions

The UPLC analytical conditions were as follows: the column used was Waters ACQUITY UPLC HSS T3 C18 (1.8 µm, 2.1 mm × 100 mm); column temperature was maintained at 40 °C; flow rate was set at 0.4 mL/min; injection volume was maintained at 2 μL; solvent system consisted of water (0.1% formic acid) and acetonitrile (0.1% formic acid); gradient program was initiated at 95:5 *v*/*v* at 0 min, followed by a shift to 10:90 *v*/*v* at 11.0 min, was maintained at 10:90 *v*/*v* until 12.0 min and then reverted to 95:5 *v*/*v* at 12.1 min and maintained at 95:5 *v*/*v* until 14.0 min.

#### 2.8.3. Mass Spectrometry Conditions

The mass spectrometry was equipped with an electrospray ionization source, with the samples analyzed in both positive and negative ionization modes. The parameters for positive (+) and negative (−) ESI modes were set as follows: capillary voltage at 2.5 kV (ESI+) and 1.5 kV (ESI-), fragmentation voltage at 135 V, a drying gas flow rate of 8 L/min at 325 °C, sheath gas flow rate of 11 L/min at 325 °C, and nebulizer pressure set at 4.0 psi.

#### 2.8.4. Non-Targeted Metabolomics Data Processing

The original LC–MS data files were converted to mzML format using ProteoWizard software (version 3.0). Subsequently, peak extraction, alignment, and retention time correction were carried out using the XCMS program. The peak area was corrected using the “SVR” method, and peaks with a detection rate below 50% in each sample group were excluded. Metabolites were identified based on laboratory’s self-built database MWDB (Metware Biotechnology Co., Ltd., Wuhan, China) and the integrated public database includingMetlin (https://metlin.scripps.edu (accessed on 16 June 2022)), HMDB (https://hmdb.ca/ (accessed on 16 June 2022)), KEGG (https://www.kegg.jp/ (accessed on 16 June 2022)), Mona (https://mona.fiehnlab.ucdavis.edu/ (accessed on 16 June 2022)), and MassBank (http://www.massbank.jp/ (accessed on 16 June 2022)), and metDNA (Metabolite identification and Dysregulated Network Analysis). Identification scoring was composed of precursor ions Q1 score, retention time (RI) score, and MS/MS similarity score, among which, Q1 allowed for an error of 25 ppm; MS/MS allowed for an error of 50 ppm, and RI allowed for an error of 60 s [16]. A total score greater than 0.85 was considered a positive identification. Identified metabolites underwent Principal Component Analysis (PCA), Orthogonal Partial Least Squares Discriminant Analysis (OPLS-DA), and S-plot analysis by SIMCA (version 14.1). To avoid overfitting, a permutation test (200 permutations) was conducted. For two-group analysis, the method referred to by Zhang et al. [17] was slightly modified to identify differential metabolites, and the screening criteria were Variable Importance in Projection (VIP) (VIP ≥ 1), covariance (p) (|p (1)| > 0.1), and correlation (pcorr) (|pcorr (1)| > 0.5). VIP values were extracted from the OPLS-DA result, and |p (1)| and |pcorr (1)| were extracted from S-plot.

### 2.9. Statistical Analysis

Each experiment was repeated 5 times. The data were expressed as mean value ± standard deviation (*n* = 5), and plotted with GraphPad Prism (version 9) and Origin (version 2021). The obtained results were subjected to statistical analysis using one-way analysis of variance (ANOVA) with Duncan post-test (*p* < 0.05) through IBM SPSS (version 20.0). The Mantel test was performed using ChiPlot (https://www.chiplot.online/ (accessed on 25 July 2024)). Correlation map between differential metabolites and antioxidant activity was performed with R software (v.4.2.2) package “corrplot” (v.0.92) and “ggplot2” (v3.4.2) in Hiplot Pro (https://hiplot.com.cn/ (accessed on July 25th, 2024)), a comprehensive web service for biomedical data analysis and visualization.

## 3. Results

### 3.1. Taste Characteristics

**Figure 1:**
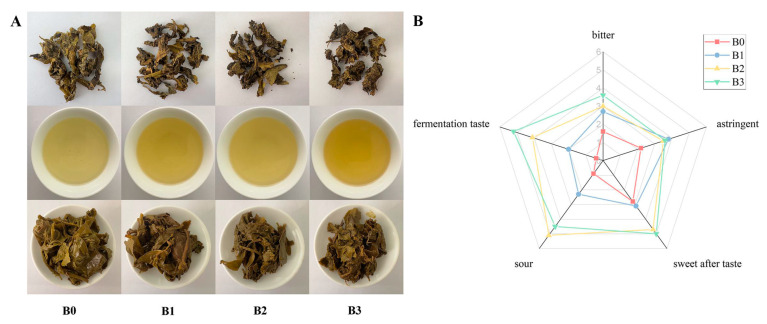
Changes in taste characteristics of Bulang pickled tea during fermentation. (Note: (**A**) appearance of dry teas, tea infusions, and brewed tea leaves; (**B**) radar plot of the taste properties).

The taste characteristics of Bulang tea’s different fermentation degree was determined based on traditional taste evaluations, as depicted in Figure 1A,B. Figure 1A presents the appearance and infusions of dry teas, brewed tea leaves, and tea infusions of Bulang pickled tea with different fermentation durations. B0 tea samples displayed dark green leaves and a greenish–yellow soup color. The appearance and infusions turned brown significantly as fermentation proceeded. Pickled tea fermented for three months (B3) had the darkest greenish–yellow infusion and green leaves. The taste properties of Bulang pickled tea are shown in Figure 1B, including five attributes (bitter, astringent, sweetness aftertaste, sour, and fermentation taste). The shape of the Bulang pickled tea attribute map varied with fermentation duration, indicating that the taste of Bulang pickled tea significantly changed during fermentation. Unfermented tea (B0) had the lowest scores for five attributes. With the extension of fermentation time within three months, the intensities of five tastes increased to different degrees. Overall, Bulang pickled tea fermented for three months (B3) exhibited the highest taste value, with fermentation taste at point 5.2, sourness at point 4.5, sweetness aftertaste at point 5.0, astringency at point 3.6, and bitterness at point 3.6 on a six-point intensity scale. It is worth noting that Bulang pickled tea with two-month fermentation (B2) showed a higher intensity of sourness compared with B3. These results indicate that the sensory quality of Bulang pickled tea changed significantly with fermentation, and a three-month fermentation period confers pickled tea with a distinctively better sweetness and fermentation taste.

### 3.2. Culturable Microbes Counting

**Figure 2:**
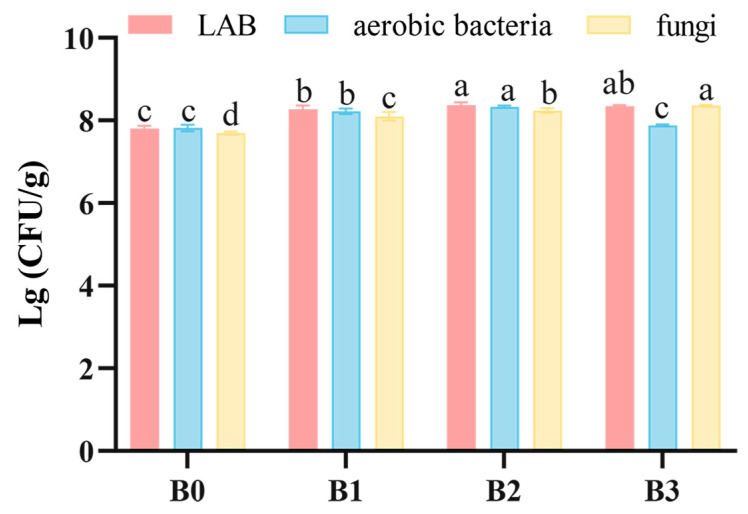
Count results of culturable microbes. (Note: The difference letters indicate significant differences between tea samples fermented with different durations (*p* < 0.05)).

The viable counts of total aerobic bacteria, LAB, and fungi in Bulang pickled tea through fermentation were presented in Figure 2. The viable counts of LAB, total aerobic bacteria, and fungi in Bulang pickled tea significantly increased with fermentation time (*p* < 0.05) up to two months. At 2 months of fermentation, the colony counts of LAB, aerobic bacteria, and fungi were 8.37 ± 0.07 lg (CFU)/g, 8.33 ± 0.03 lg (CFU)/g, and respectively 8.24 ± 0.06 lg (CFU)/g. When fermented for 3 months, Bulang pickled tea still harbored higher LAB and fungi. Interestingly, a significant decrease in total aerobic bacteria was observed during the period from 2 to 3 months (*p* <0.05). These above results indicate that both LAB and fungi are involved in entire fermentation for three months while total aerobic bacteria are mainly associated with early fermentation. Unban, Khatthongngam, Pattananandecha, Saenjum, Shetty and Khanongnuch [8] reported that similar results of viable counts of these microorganisms were observed in *Miang* and viable cell numbers of LAB, yeast, and total aerobic bacteria tended to be stable after three months of fermentation, highlighting the robustness in the microbial community during the first three months of pickled tea fermentation. However, in contrast to *Miang* and *Laphet*, Bulang pickled tea has shown a higher microbial load than that of those, and this phenomenon may be related to the greater microbial counts at the initial point of fermentation, suggesting that the autochthonous microbial populations and/or pretreatment practice may shape the microbial community in pickled tea during fermentation.

### 3.3. Physiochemical Parameters

**Figure 3:**
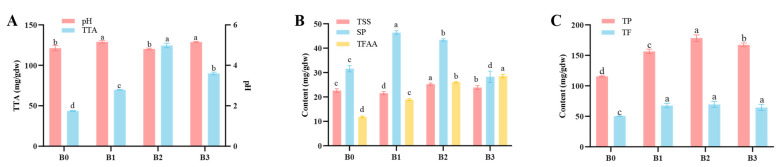
Changes in chemical contents of Bulang pickled tea during fermentation. (Note: (**A**) variations in pH and total titratable acidity (TTA); (**B**) content of total soluble sugar (TSS), soluble protein (SP), and total free amino acid (TFAA); (**C**) total phenol (TP) and total flavonoid (TF) contents. The results were expressed as mean values ± standard deviation. The difference letters indicate significant differences between tea samples fermented with different duration (*p* < 0.05)).

The variations in pH and TTA during Bulang pickled tea are illustrated in Figure 3A. Initially, the pH showed an upward trend, followed by a decline and, eventually, a subsequent rise, fluctuating within the range of 4.81–5.16. Conversely, TTA exhibited an initial increase, peaking at 124.56 mg/gdw in the second month of fermentation and then decreasing to 90.05 mg/g dw by the end of the fermentation period. The changes in TTA in the present study align with those of *Miang* prepared only anaerobically in the absence of aerobic fermentation [8], and therefore, non-filamentous fungi, such as LAB and yeast, contribute to the acid production in pickled tea. Interestingly, the observed disparity between pH and total titratable acidity content was shown, which may be due to the buffering effects of the tea base, as reported by Cvetkovic et al. [18]. Notably, pickled tea distinguishes itself from other fermented teas by its pronounced sour taste, intensifying with prolonged fermentation.

The fluctuations in the content of total soluble sugar (TSS), soluble protein (SP), and total free amino acid (TFAA) throughout the fermentation of Bulang pickled tea were observed (Figure 3B). The content of TSS content showed a fluctuating pattern, initially decreasing, followed by an increase and subsequent decrease. It attained a peak value of 25.24 ± 0.43 mg/gdw (*p <* 0.05) in the second month of fermentation, subsequently experiencing a significant decrease (*p <* 0.05). Similarly, the SP content displayed an ascending then descending trend during fermentation, peaking significantly at 46.38 ± 0.76 mg/gdw (*p <* 0.05) in the first month and then decreasing significantly (*p <* 0.05) to a minimum of 28.25 ± 2.35 mg/gdw in the third month. In contrast, the TFAA content significantly increased throughout the fermentation process (*p <* 0.05). The initial TFAA was 11.80 ± 0.37 mg/gdw before fermentation, reaching a maximum of 28.57 ± 0.76 mg/gdw in the third month of fermentation.

The constituents such as TSS, SP, and TFAA play pivotal roles in determining the flavor profile of tea infusion. The initial decrease in TSS content during the early stages of Bulang pickled tea fermentation may be attributed to the vigorous growth and reproduction of microorganisms. Compared with the decline of SP content during Pu-erh tea fermentation, the rise in the SP content in Bulang pickled tea was found, and this could be interpreted by the release of proteins trapped in tea leaves through the enzymes produced by microorganisms [19]. Moreover, the proliferation of microorganisms during fermentation may lead to the secretion of numerous proteins [20,21], contributing to the observed increase in soluble proteins. The subsequent decline in soluble protein during the later fermentation stage could be attributed to the utilization of soluble protein as a nitrogen source for microbial growth.

### 3.4. Phytochemical Contents

The alterations in total phenols (TP) and total flavonoids (TF) content during the fermentation process of Bulang pickled tea are depicted in Figure 3C. The content of TP had an initial increase followed by a decrease, while TF significantly increased at the first month of fermentation (*p* < 0.05) and then stabilized. Both total phenols and total flavonoids content reached their peak values in the second month of fermentation, measuring 178.38 ± 5.26 mg/gdw and 69.59 ± 4.91 mg/gdw, respectively. The acidic environment caused by the organic acids during the fermentation process may also impede the degradation of phenolics by influencing the activity of enzymes responsible for breaking down flavonoids and phenols in the fermentation solution [22].

### 3.5. Antioxidant Activity

**Figure 4:**
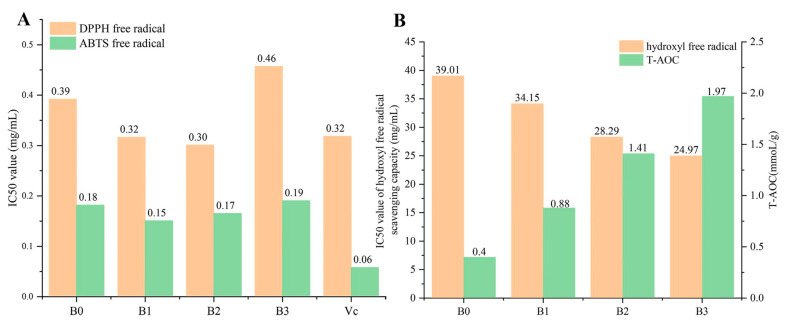
Changes in the antioxidant activity of Bulang pickled tea during fermentation. (Note: (**A**) ABTS and DPPH-free radical scavenging ability; (**B**) T-AOC and hydroxyl-free radical scavenging ability).

The antioxidant activity of Bulang pickled tea with different fermentation time are shown in Figure 4. Hydroxyl radicals are one of the reactive oxygen species, while DPPH and ABTS-free radicals are stable radicals for hydroxyl radicals and superoxide anion [23]. The determination of the scavenging capacity of DPPH and ABTS radicals is considered to be a simple method for measuring antioxidant activity. T-AOC can be determined by reducing the power of ferric since iron ions can be reduced to decrease the generation of hydroxyl radicals, thereby reducing reactive oxygen species [24]. The IC50 value of ABTS and DPPH-free radical scavenging capacities decreased slightly at first and then increased with the extension of fermentation time (Figure 4A). These results indicated that the ABTS and DPPH-free radical scavenging capacities of Bulang pickled tea fermented for 1 and 2 months were increased to some extent compared with unfermented tea, and the DPPH-free radical scavenging capacities of these two fermentation stages were similar to VC. In this study, the overall change trend of the free radical scavenging ability of ABTS was consistent with the change trend of total phenol and total flavone contents through three months of fermentation, indicating that the enhancement of the free radical scavenging ability of ABTS might be related to the increase in total polyphenol and total flavone contents during fermentation. The pattern of DPPH fluctuation was similar to that of total polyphenol and total flavone content fluctuation during fermentation for the first two months, and at the third month of fermentation, DPPH inhibition of pickled tea dropped to reach the highest IC50 value with the sight reduction in total polyphenol and total flavone concentration. T-AOC and hydroxyl-free radical scavenging capacity increased continuously throughout the fermentation process (Figure 4B). The tea fermented for three months (B3) had the lowest IC50 value of hydroxyl-free radical scavenging capacity (24.97 mg/mL) and the highest total antioxidant capacity (1.97 mmol/g). The previous study has found that polysaccharides from *Schisandra sphenanthera* fruit significantly increased after lactic acid bacteria (*Lactobacillus plantarum*) fermentation [25], which may be the reason for the increase in T-AOC and hydroxyl-free radical scavenging capacity. It is worthy to note that the inconsistent alteration between antioxidant activity measures and total polyphenols and flavonoid was observed, suggesting that fermentation led to the complicated impact on the bioactivity of Bulang pickled tea due to the modified composition of polyphenols via a switch from antioxidant polyphenolic substances to those without antioxidant activities.

### 3.6. Mantel Test Analysis of Culturable Microbes, Physiochemical Parameters, Physiochemical Parameters, and Antioxidant Measures

**Figure 5:**
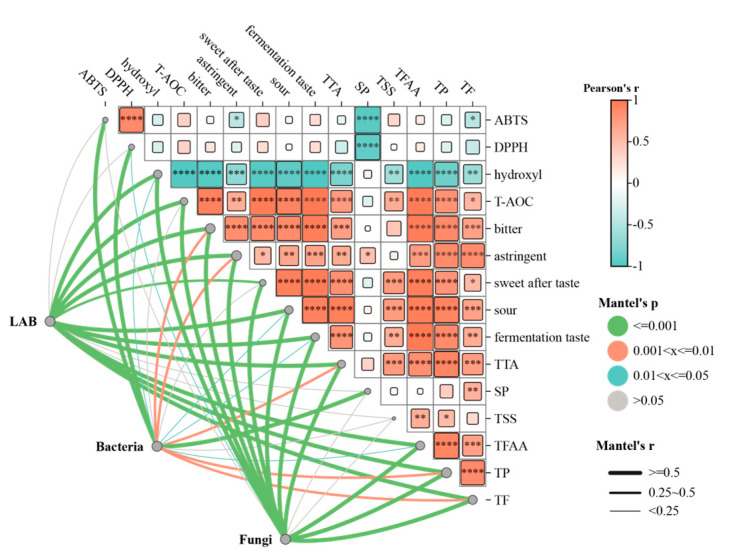
Mantel test analysis of culturable microbes, taste, physiochemical, and antioxidant measures. (Note: The lines represented the correlation, and the thickness of the lines represented the magnitude of the correlation, which was drawn with mantel ’r (absolute value of r). The heat map represents the correlation between chemicals and antioxidant activity; Pearson correlation coefficient ranges from −1 to 1; r < 0 is negative correlation; r > 0 is positive correlation. * indicates a significant difference, * *p* ≤ 0.05, ** *p* ≤ 0.01, *** *p* ≤ 0.001, **** *p* ≤ 0.0001).

The mantel test was employed to further investigate the ralationship between culturable microbes and taste characteristics, physiochemical parameters, and antioxidant measures (Figure 5). Fermentation parameters, including TTA, TFAA, TP, and TF, were significantly correlated with culturable LAB and fungi (mantel’s r ≥ 0.5, mantel’s *p* ≤ 0.001), indicating that these factors had a significant influence on these microbial communities. Furthermore, the driving effects of these factors on these two communities were generally the same, suggesting the synergistic interaction of them. Then, LAB and fungi were also shown to have a substantial correlation with taste attributes, including bitterness, astringency, sweetness after taste, sourness, and fermentation taste (mantel’s r ≥ 0.5, mantel’s *p* ≤ 0.001). The increased production of organic acids, TP, and TF could be the intermediate components through which LAB and fungi modulate the taste development of Bulang pickled tea [26]. This indicated that modified tea phytochemicals and microbial metabolites during fermentation contribute to the taste of fermented tea [27,28]. However, the relationship between the antioxidant measures and different microorganisms was dependent on the type of microorganisms. For example, LAB and fungi were correlated with T-AOC and hydroxyl-free radical scavenging capacity (mantel’s r ≥ 0.5, mantel’s *p* ≤ 0.001); the lower IC50 values mean the higher activity of DPPH, ABTS, and hydroxyl-free radical of pickled tea samples, and the higher concentration values presents the higher T-OAC. This may be the result of the increased release of phenolic compounds during the fermentation process of Bulang pickled tea due to hydrolyzing phenolic glycosides and the breakdown of the cell wall through enzymes produced by LAB and fungi [29]. The positive correlations of T-AOC and hydroxyl-free radical scavenging capacity with TP and TF, as well as LAB and fungi, support this assumption (Figure 5). Furthermore, ABTS and DPPH-free radical scavenging ability were strongly correlated with total aerobic bacteria, indicated the role of aerobic bacteria involved in up-regulation of these two antioxidant parameters. The correlation analysis shows that soluble proteins were positively correlated with ABTS and DPPH-free radical scavenging ability, supporting the previous evidence showing that some proteins or pep-tides generated by microbiota residing in fermented foods contribute to the capacity to scavenge ABTS and DPPH-free radicals [30]. The above results indicate that different functional strategies may be present in different microbial groups in Bulang pickled tea.

### 3.7. Metabolomics Analysis of Changes in Non-Volatile Metabolites

#### 3.7.1. Analysis of Non-Volatile Metabolites

**Figure 6:**
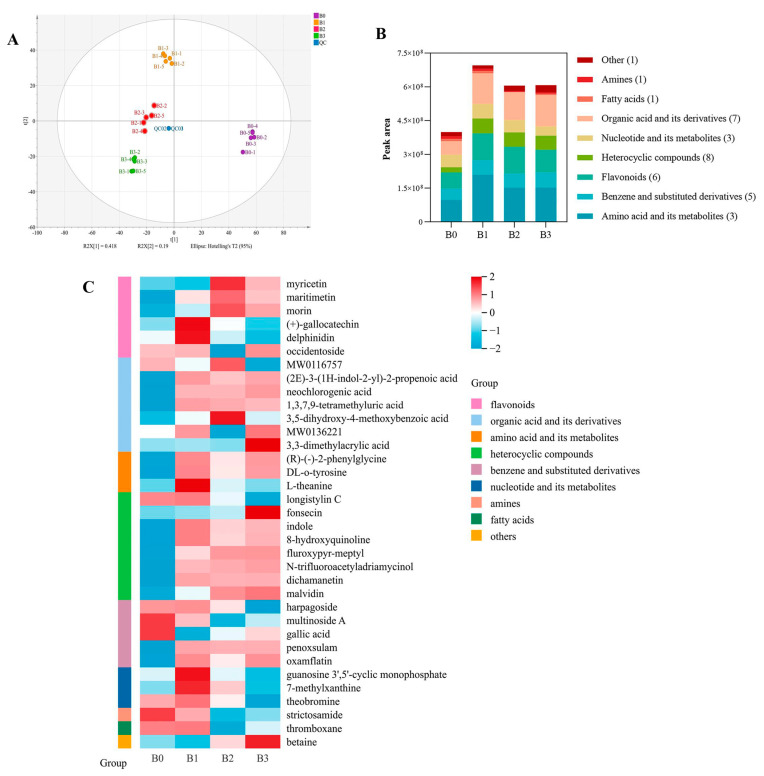
Analysis of changes in non-volatile metabolites. (Note: (**A**) PCA score chart of non-volatile metabolites; (**B**) differential metabolite peak area map; (**C**) differential metabolite heat map. MW0116757 means “1-[(3,5-Dimethyl-1,2-oxazol-4-yl) sulfonyl] piperidine-4-carboxylic acid”; MW0136221 means “6-(2-[6-carboxy-5-(2,4-dihydroxyphenyl)-3-methylcyclohex-2-en-1-yl]-3-hydroxy-5-[6-hydroxy-7-(3-methylbut-2-en-1-yl)-1-benzofuran-2-yl] phenoxy)-3,4,5-trihydroxyoxane-2-carboxylic acid”; QC means Quality Control Samples).

In both positive and negative ion modes, a total of 1246 and 1152 metabolites were respectively identified. The total ion chromatograms of Bulang pickled tea samples in both positive and negative modes were presented in Figures S1–S3. As shown in Figure S3, the superposition of total ion chromatogram for all quality control samples in both positive and negative modes was determined, suggesting good stability, reproducibility, and consistency of UHPLC-QTOF-MS. The Principal Component Analysis (PCA) results depicted in Figure 6A revealed distinct separation between groups and tight aggregation within groups for Bulang pickled tea with varying fermentation times. This indicates substantial differences in the non-volatile metabolites of Bulang pickled tea fermented with different periods, with relatively minor differences within each group. The PCA results demonstrated that the first two principal components elucidated 41.8% and 19.0% of the total variance. The Orthogonal Partial Least Squares Discriminant Analysis (OPLS-DA) model, utilizing R_2_X, R_2_Y, and Q_2_ as forecasting parameters, exhibited stability, absence of overfitting, and high prediction accuracy (refer to Figure S4). A Q_2_ value greater than 0.5 indicates a valid model, while Q_2_ surpassing 0.9 signifies an excellent model. These results underscore the significant impact of fermentation duration on the composition of non-volatile metabolites in Bulang pickled tea, emphasizing that the distribution of metabolites undergoes notable changes with increasing fermentation time. The unfermented samples predominantly occupy the left quadrant, gradually transitioning to the right quadrant with prolonged fermentation time, indicating that metabolite changes become more pronounced with increased fermentation duration.

#### 3.7.2. Dynamic Changes in Differential Non-Volatile Metabolites

In order to find out the potential marker compounds that cause differences in the Bulang pickled tea fermentation process, a combination of Variable Importance in Projection (VIP), covariance (p), and correlation (pcorr) was employed. Based on the rule of VIP and *p*, a total of 35 differential metabolites were identified in the fermentation process of Bulang pickled tea under both positive and negative ion modes (Tables S1 and S2). These differential non-volatile metabolites, including nine categories (Figure 6B), eight heterocyclic compounds, seven organic acids and their derivatives, six flavonoids, five benzene and substituted derivatives, three amino acids and their derivatives, three nucleotide and its metabolites, one fatty acid, one amine, and one other. The diversity of these metabolites indicates that the non-volatile components undergo complex and dynamic biochemical transformation during the fermentation of Bulang pickled tea while showing a general tendency of increase with fermentation.

A heat map was applied to visualize the dynamic changes in the potential marker compounds during the fermentation of Bulang pickled tea (Figure 6C). Flavonoids and amino acids have a significant impact on the formation of tea quality, while fatty acids and organic acids are the main metabolites of microbial fermentation. Among these differential flavonoids during Bulang pickled tea, myricetin, maritimecin, morin, and occidentoside increased with fermentation while delphinidin and (+)-gallocatechin decreased, which may be related to the net result of the production and breakdown of targeted phenols via the complex microbiome and various enzyme reactions during fermentation. A study has found that the myricetin content in tea inoculated with either of seven fungus strains derived from Pu-erh tea significantly increased [31], leading to the hypothesis that fungi fermentation improves the accumulation of myricetin in Bulang pickled tea. Then, six out of seven differential organic acids and their derivatives in Bulang pickled tea increased with fermentation, suggesting that the anaerobic fermentation process, characterized by the activities of lactic acid bacteria and other microorganisms, results in the production of numerous organic acids and metabolites.

The overall increasing trend observed in differential metabolites falling into the category of amino acids and their metabolites during the fermentation of Bulang pickled tea paralleled with the changes in the content of total free amino acids, confirming that free amino acids production was caused by the proteinaceous degradation. The increase in theanine during the early stage of fermentation may be related to up-regulatory effect of organic acids on its biosynthesis [32], and the decline in the following period could be due to the degradation of microbial fermentation into glutamic acid and ethylamine overpowering the production [33].

The heterocyclic compounds accounted for the largest portion of the total differential metabolites and showed an overall increasing trend during the fermentation of Bulang pickled tea. During the fermentation of Pu-erh tea, heterocyclic compounds generation was mainly associated with microbial metabolism [34]. It is also evidenced that inoculation with *Lactobacillus plantarum* or *Aspergillus oryzae* converts anthocyanin glycosides malvidin-3,5-diglycosides into anthocyanin aglycones malvidin of black carrot extract [35]. These results interpret the role of microorganisms influencing the changes in heterocyclic compounds during Bulang pickled tea fermentation. Benzoic acid derivatives, such as gallic acid, are predominant in teas, and gallic acid contributes to the sweetness aftertaste of green tea infusion [36], and its increase in Bulang pickled tea could be a reason for the enrichment of sweetness aftertaste. The presence of these fatty acids in Bulang pickled tea contributes to its overall aroma profile, adding complexity and richness to the sensory characteristics of the fermented tea.

### 3.8. Correlation Analysis of Non-Volatile Metabolites and Antioxidant Activity

**Figure 7:**
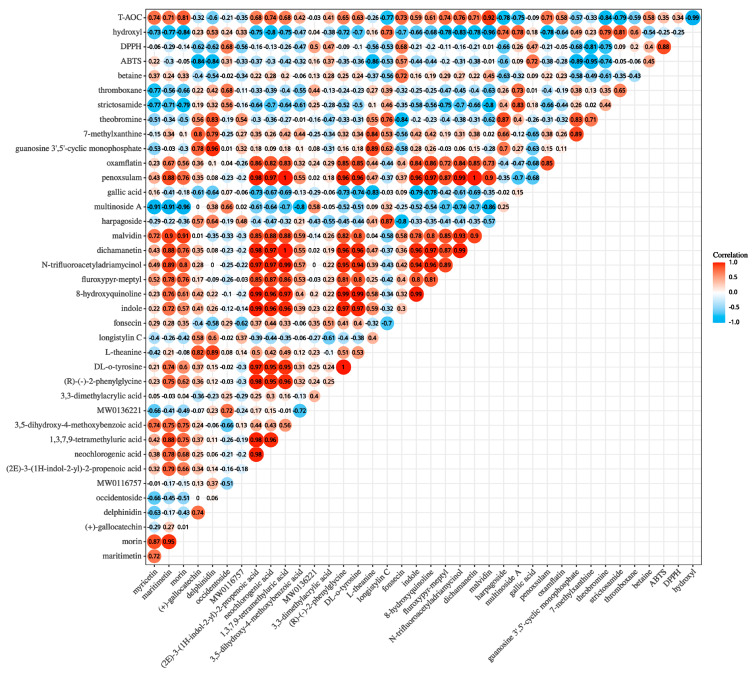
Correlation map of taste characteristics and antioxidant activity. (Note: MW0116757 means “1-[(3,5-Dimethyl-1,2-oxazol-4-yl) sulfonyl] piperidine-4-carboxylic acid”; MW0136221 means “6-(2-[6-carboxy-5-(2,4-dihydroxyphenyl)-3-methylcyclohex-2-en-1-yl]-3-hydroxy-5-[6-hydroxy-7-(3-methylbut-2-en-1-yl)-1-benzofuran-2-yl] phenoxy)-3,4,5-trihydroxyoxane-2-carboxylic acid”. The color of the circle indicates the correlation coefficient, and the size of the circle indicates statistical difference. The red color circle indicates positive (0 ≤ r ≤ 1) correlation, and the blue color circle indicates negative (−1 ≤ r ≤ 0) correlation).

Pearson correlation analysis method was conducted to evaluate the associations between potential markers and antioxidant activities (DPPH, ABTS, hydroxyl, T-AOC) (Figure 7). Based on Pearson correlation coefficient |r| > 0.8, *p <* 0.05, differential metabolites with greater influence on antioxidant activities were screened, respectively. Overall, antioxidant activity was positively correlated with nine potential markers, including three flavonoids (delphinidin, morin, (+)-gallocatechin), two heterocyclic compounds (N-trifluoroacetyladriamycinol and malvidin), two nucleotide and its metabolites (guanosine 3′,5′-cyclic monophosphate, 7-methylxanthine), one amino acid and its metabolites (L-theanine), and one organic acid and its derivative (neochlorogenic acid) (Figure 7). Therefore, flavonoids, heterocyclic compounds, and nucleotides and their metabolites were the metabolites most associated with antioxidant activities in Bulang pickled tea. As mentioned above, the lower IC50 values mean the higher activity of DPPH, ABTS, and hydroxyl-free radicals of pickled tea samples, and the higher concentration values present the higher T-OAC. ABTS-free radical scavenging ability was significantly positively correlated with five metabolites consisting of 7-methylxanthine, guanosine 3′,5′-cyclic monophosphate, L-theanine, (+)-gallocatechin, and delphinidin. The 7-methylxanthin was positively related to the effect on DPPH-free radical scavenging ability. Additionally, malvidin, morin, N-trifluoroacetyladriamycinol, and neochlorogenic acid were positively correlated with hydroxyl radical scavenging ability, and malvidin and morin were positively related with T-AOC. Given that morin, malvidin, and 7-methylxanthine were all significantly positively correlated with two antioxidant indicators, this indicated that they may possess more prominent antioxidant activities. Three associated flavonoids included (+)-gallocatechin, morin, and delphinidin, which have been shown to have strong antioxidant activity [37]; morin can exhibit antioxidant activity through inhibiting xanthine oxidase, inducing gene expression of enzymes involved in antioxidant response and forming stable complexes with transition metal ions [38]. Moreover, morin and delphinidin might potentially exert anti-inflammatory [39] and anticancer effects [40] due to their antioxidant properties. Heterocyclic compounds have shown a wide range of biological functions including antioxidant activity [41]. The results obtained here indicated that heterocyclic compounds help to enhance antioxidant activity, which was consistent with previously published data [42], suggesting that fermentation has the potential to enrich the heterocycles with antioxidant activity. A nucleotide and its metabolites 7-methylxanthine, one metabolite derived from caffeine, was present in tea plant and products [43,44] has shown nitric oxide radical scavenging capacity [45]. A positive correlation was found between neochlorogenic acid and hydroxyl radical scavenging ability in the present study, indicating that neochlorogenic acid had the ability to remove hydroxyl-free radicals [46] and could be a potential antioxidant. These findings suggest that Bulang pickled tea may have potential benefits in alleviating oxidative damage and anti-inflammatory and anticancer effects.

## 4. Conclusions

In the present study, the dynamics of physicochemical properties, antioxidant activity, and non-volatile metabolites in Bulang pickled tea during fermentation were investigated. Fermentation duration showed an important effect on the physicochemical characteristics, antioxidant function, and changes in non-volatile metabolites of Bulang pickled tea, and the results demonstrated that Bulang pickled tea with a fermentation period of 2 months presented a stronger sour taste and antioxidant activity. Metabolomics analysis revealed that nine metabolites, including flavonoids, heterocyclic compounds, and nucleotide and its metabolites, were significantly related to antioxidant activities. Given that morin, malvidin, and 7-methylxanthine are all significantly positively correlated with two antioxidant indicators, this indicates that they may possess more prominent antioxidant activities. These metabolites may serve as indicators for the fermentation process and contribute to understanding the quality formation in Bulang pickled tea. The present study provides a solid base for the key chemicals associated with quality characteristics as well as the microorganisms involved in their formation and offers rational insights for optimizing the manufacturing process in pickled tea.

## Data Availability

The original contributions presented in this study are included in this article/Supplementary Material. Further inquiries can be directed to the corresponding authors.

## Supplementary Materials

The following supporting information can be downloaded at https://www.mdpi.com/article/10.3390/foods14050878/s1, Figure S1. Representative TIC of Bulang pickled tea sample with different fermentation times in the positive ion mode. Figure S2. Representative TIC of Bulang pickled tea sample with different fermentation times in the negative ion mode. Figure S3. The overlap of TIC of QC sample in the positive and negative ion modes. (A) positive ion mode, (B) negative ion mode. Figure S4. OPLS-DA scores chart and verification chart of different comparison groups. Table S1: Differential metabolites of Bulang pickled tea with different fermentation time; Table S2: VIP, p(1), pcorr(1) value of 35 differential metabolites of Bulang pickled tea with different fermentation time.
