# Supplementary material for: Dynamics Changes in Physicochemical Properties, Antioxidant Activity, and Non-Volatile Metabolites During Bulang Pickled Tea Fermentation
Source: Foods. 2025 Mar 4;14(5):878. doi: 10.3390/foods14050878 (PMC11898713; doi:10.3390/foods14050878)
Supplement: Supplementary file 1 [file foods-14-00878-s001.zip › foods-3500270-supplementary.pdf]

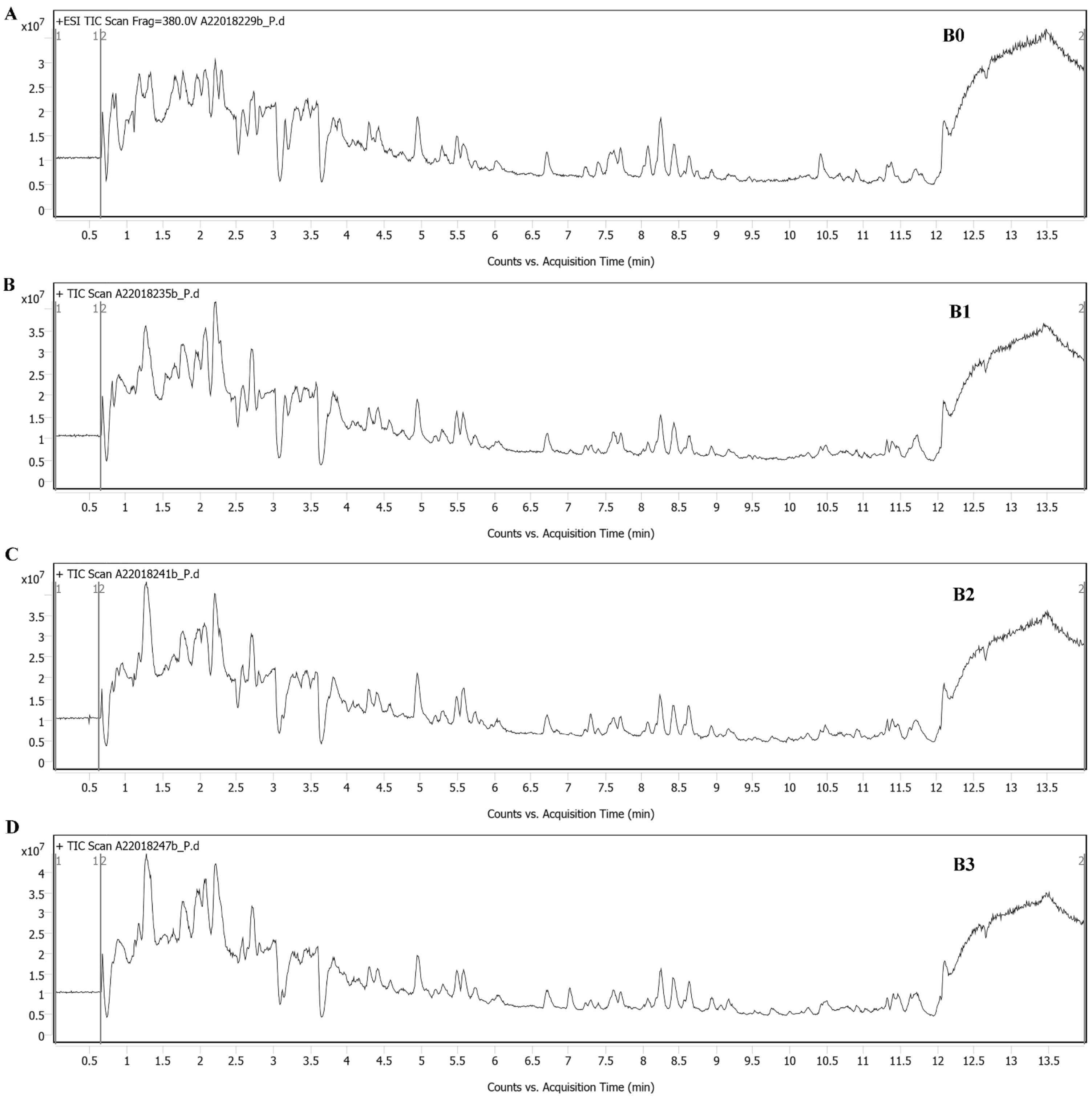

Figure S1. Representative TIC of **Bulang pickled tea** sample with different fermentation times in the positive ion mode.

Note: B0, B1, B2 and B3 respectively represent Bulang pickled tea fermented for 0, 1, 2 and 3 months.

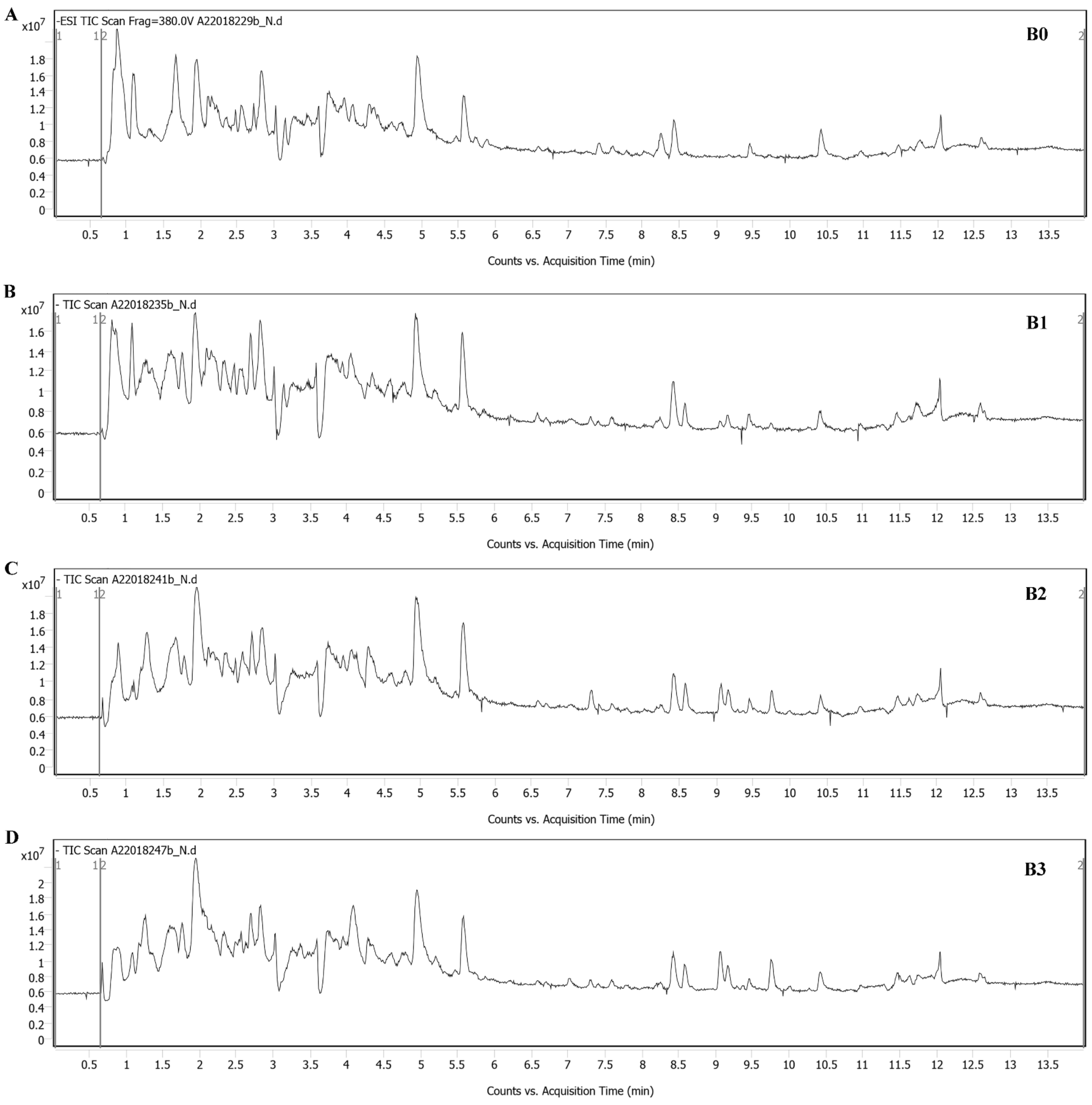

Figure S2. Representative TIC of Bulang pickled tea sample with different fermentation times in the negative ion mode.

Note: B0, B1, B2 and B3 respectively represent Bulang pickled tea fermented for 0, 1, 2 and 3 months.

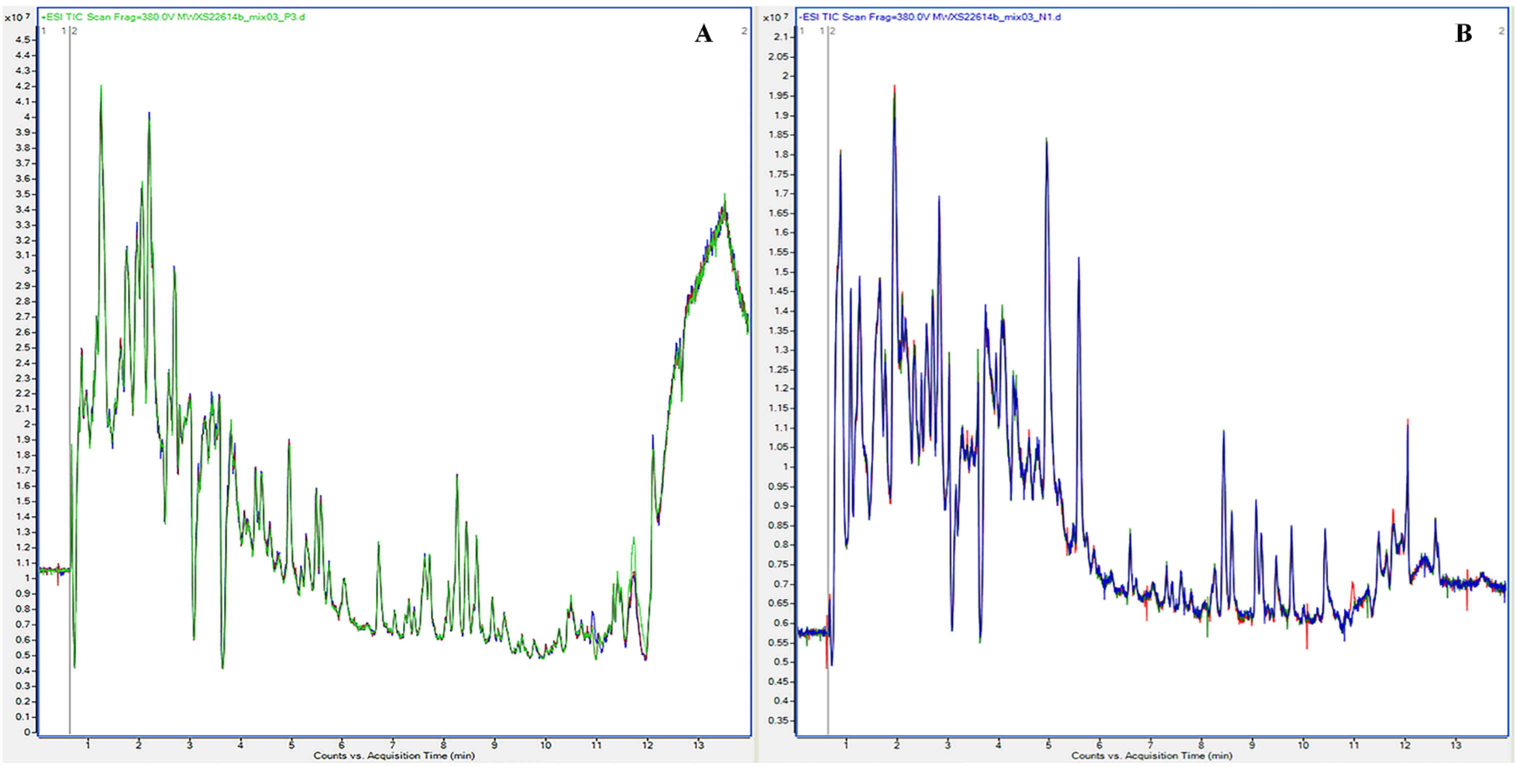

Figure S3. The overlap of TIC of QC sample in the positive and negative ion modes. (A) positive ion mode, (B) negative ion mode.

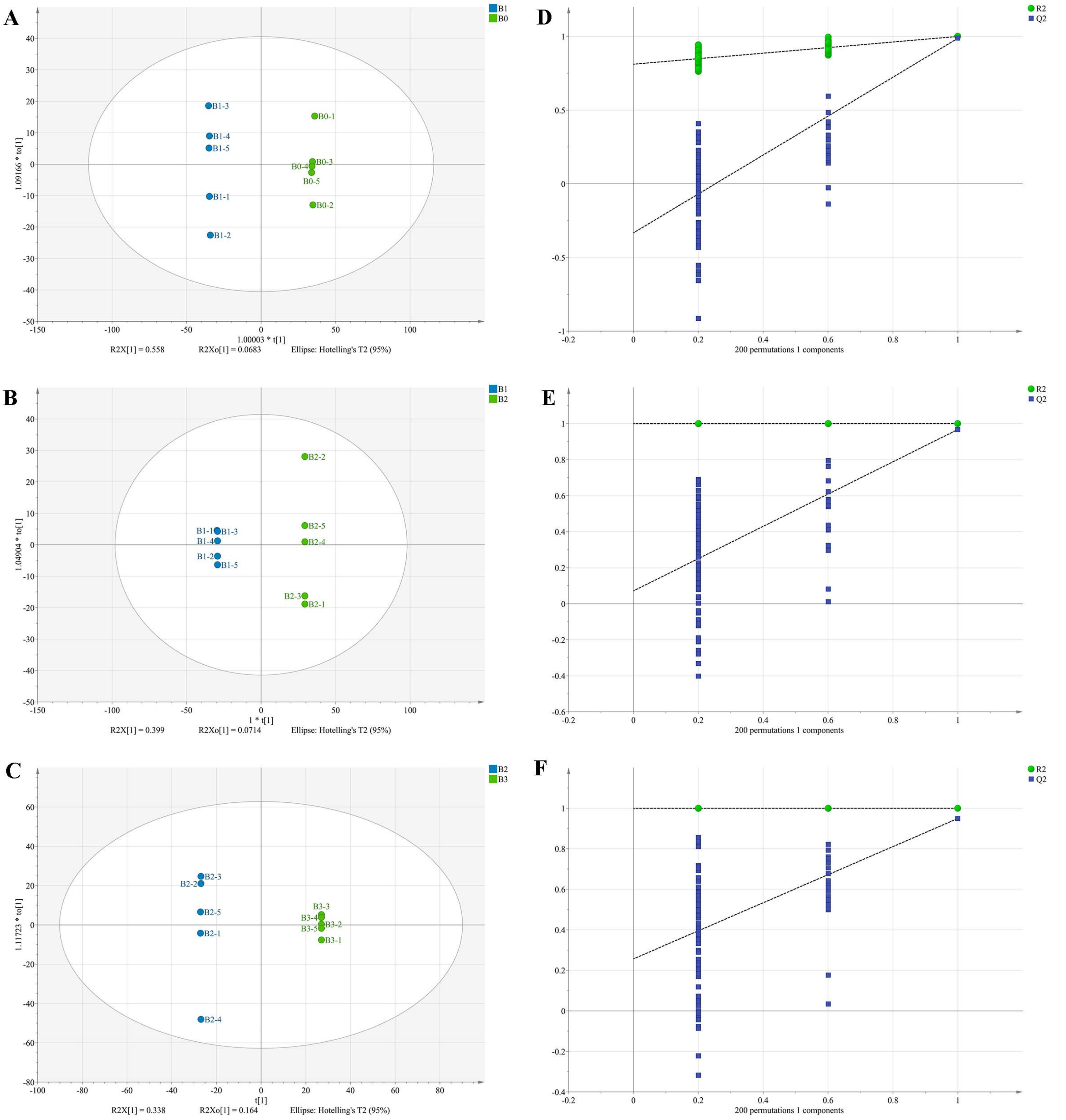

Figure S4. OPLS-DA scores chart and verification chart of different comparison groups

Note: B0, B1, B2 and B3 respectively represent Bulang pickled tea fermented for 0, 1, 2 and 3 months, A-C is the scores chart and D-F is the verification chart.

Table S1. Differential metabolites of Bulang pickled tea with different fermentation time

ID	Compounds	Formula	Class	Precursor (g/mol)	Mass (g/mol)	RT (min)	Adduct	score	CAS	PubChem CID	B0	B1	B2	B3
MW0126595	strictosamide	C26H30N2O8	Amines	565.1545	498.2002	3.2304	M-2H+3Na	0.9233	23141-25-5	10345799	11360759.86	9447046.446	4073110.626	5803774.2
MEDP1126	(R)-(-)-2-phenylglycine	C8H11NO	Amino acid and its metabolites	120.0823	137.0841	1.7815	M+H-H2O	0.9489	56613-80-0	70134	24593356.07	62597169.56	54301405.22	61202569
MEDN0050	L-theanine	C7H14N2O3	Amino acid and its metabolites	175.1081	174.1004	0.8802	M+H	0.9687	3081-61-6	439378	46133176.25	111724093.3	68303581.07	57736969
MW0106523	DL-o-tyrosine	C9H11NO3	Amino acid and its metabolites	166.0862	181.0739	1.7814	M+H	0.9712	150-30-1	91482	10583395.49	35703231.01	29518612.39	34270926
MW0009353	penoxsulam	C16H14F5N5O5S	Benzene and substituted derivatives	523.0861	483.0636	2.4926	M+Na+NH3	0.851	219714-96-2	11784975	2840632.64	16215351.08	15713004.07	15795858
MW0053814	harpagoside	C24H30O11	Benzene and substituted derivatives	517.166	494.1788	4.13	M+Na	0.8621	19210-12-9	5281542	5351142.503	5440249.329	4286483.978	497248.49
MW0009295	oxamflatin	C17H14N2O4S	Benzene and substituted derivatives	381.0263	342.0674	2.6992	M+K	0.9655	151720-43-3	5353852	3559949.842	19400652.28	15166164.83	19176613
MW0130157	multinoside A	C27H30O16	Benzene and substituted derivatives	609.1459	610.1534	3.4599	M-H	0.9147	59262-54-3	5319943	11510443.39	11271301.65	8157118.038	9891580.4
MW0007231	gallic acid	C7H6O5	Benzene and substituted derivatives	169.0147	170.0215	1.6742	M-H	0.9372	149-91-7	370	22872963.46	11053443.08	18924701.4	21433047
MW0102797	thromboxane	C20H40O	Fatty acids	269.2832	296.3079	10.2072	M+H-C2H4	0.9948	-	114873	8916325.969	10807154.05	1047046.502	6294042.1
MW0127753	(+)-gallocatechin	C15H14O7	Flavonoids	307.0817	306.074	2.5339	M+H	0.9721	970-73-0	65084	20294710.36	48882204.68	31026203.06	21041972
MW0137913	delphinidin	C15H11O7	Flavonoids	345.0813	303.0505	1.6161	M+CH3CN+H	0.8696	13270-61-6	128853	7016579.916	11577728.45	8036649.571	6293950.8
MEDL01764	maritimetin	C15H10O6	Flavonoids	285.041	286.0477	5.5769	M-H	0.8976	576-02-3	5281292	10062774.97	25371968.08	31652485	26923587
MW0055176	myricetin	C15H10O8	Flavonoids	317.031	318.0376	4.2912	M-H	0.9089	529-44-2	5281672	4675925.373	3611656.422	14376965.9	10422949
MW0108349	morin	C15H10O7	Flavonoids	301.0356	302.0427	4.9485	M-H	0.9279	480-16-0	5281670	16163986.79	24666551.55	33059264.45	30039894
MW0010635	occidentoside	C36H32O15	Flavonoids	739.141	704.1741	1.1202	M+Cl	0.9618	-	6440594	5224631.902	5109230.882	1174037.106	5542931.6
MW0124151	fluroxypyr-meptyl	C15H21Cl2FN2O3	Heterocyclic compounds	367.0977	366.0913	0.8174	M+H	0.9056	81406-37-3	54745	356320.8158	7331632.168	8857986.785	8917506.9
MW0137945	dichamanetin	C29H24O6	Heterocyclic compounds	469.1662	468.1573	2.9225	M+H	0.8743	58779-09-2	181193	609759.4679	6253750.292	6060010.859	6091966.2
MEDP1060	8-hydroxyquinoline	C9H7NO	Heterocyclic compounds	146.06	145.0528	2.2279	M+H	0.916	148-24-3	-	2827578.888	9282003.384	7846580.099	8429184.1
MEDP0453	indole	C8H7N	Heterocyclic compounds	118.0653	117.0578	2.2279	M+H	0.9322	120-72-9	798	5246681.55	15917630.31	13861410.03	14540009
MW0138905	malvidin	C17H15O7+	Heterocyclic compounds	331.0813	331.0818	5.5768	M+H	0.9329	10463-84-0	159287	1609635.353	6752110.765	9696609.349	10208415
MW0124156	fonsecin	C15H14O6	Heterocyclic compounds	579.1501	290.079	2.0545	2M-H	0.8584	3748-39-8	216328	41462.34947	227058.7303	553176.7723	3601606.6
MW0139178	N-trifluoroacetyladriamycinol	C29H30F3NO12	Heterocyclic compounds	678.1373	641.172	7.2224	M-2H+K	0.9281	-	99923	394465.9403	9482960.152	9871554.246	10161053
MW0138810	longistylin C	C20H22O	Heterocyclic compounds	447.1298	278.1671	1.4999	M+Cl+CF3COOH	0.8926	-	6446720	10616843.49	11746595.53	7344755.386	1083914.9
MW0169913	theobromine	C7H8N4O2	Nucleotide and its metabolites	181.0744	180.0647	2.0776	M+H	0.9151	83-67-0	5429	34447723.75	43186059.73	38978556.15	30103409
MEDN0161	guanosine 3',5'-cyclic monophosphate	C10H12N5O7P	Nucleotide and its metabolites	346.0542	345.0474	1.543	M+H	0.9488	7665-99-8	135398569	25327346.5	6249079.002	3383178.444	1703452.4
MW0015222	7-methylxanthine	C40H56O4	Nucleotide and its metabolites	167.0564	600.4179	1.6348	M+H	0.9558	552-62-5	68374	9428644.63	14975663.5	13087420.2	10429815
MW0116757	1-[(3,5-Dimethyl-1,2-oxazol-4-yl)sulfonyl]piperidine-4-carboxylic acid	C11H16N2O5S	Organic acid and its derivatives	311.0672	288.078	7.0238	M+Na	0.9995	697258-72-3	6486599	3699493.11	2404220.829	3857729.257	578875.17
MW0013751	3,3-dimethylacrylic acid	C5H8O2	Organic acid and its derivatives	118.0859	100.0524	2.3619	M+NH4	0.9214	541-47-9	10931	5384685.316	6637100.312	6342073.801	12247195
MW0126643	1,3,7,9-tetramethyluric acid	C9H12N4O3	Organic acid and its derivatives	225.1	224.0909	2.7075	M+H	0.9402	2309-49-1	75324	101957.9862	35009623.64	33893600.18	32335266
MW0115839	(2E)-3-(1H-indol-2-yl)-2-propenoic acid	C11H9NO2	Organic acid and its derivatives	188.0731	187.0633	2.2279	M+H	0.8828	143618-94-4	15030923	23472017.1	51270412.03	48071048.8	50352414
MW0114986	neochlorogenic acid	C16H18O9	Organic acid and its derivatives	353.0898	354.0951	2.3438	M-H	0.9164	906-33-2	5280633	10353981.98	22102436.17	22030426.59	22907488
MADN0551	3,5-dihydroxy-4-methoxybenzoic acid	C8H8O5	Organic acid and its derivatives	183.0299	184.0372	2.8987	M-H	0.903	4319-02-2	78016	874481.9531	2879545.842	5447814.807	2604565.3
MW0136221	6-{2-[6-carboxy-5-(2,4-dihydroxyphenyl)-3-methylcyclohex-2-en-1-yl]-3-hydroxy-5-[6-hydroxy-7-(3-methylbut-2-en-1-yl)-1-benzofuran-2-yl]phenoxy}-3,4,5-trihydroxyoxane-2-carboxylic acid	C39H40O14	Organic acid and its derivatives	731.2372	732.2418	2.377	M-H	0.9732	-	131832611	31881259.47	15489893.37	3566669.958	16594176
MEDP0039	betaine	C5H11NO2	Other	118.0864	117.079	0.8068	M+H	0.9542	107-43-7	247	15494187.35	15481665.49	24595739.51	32497735

Notes: ID: indicates the substance number; Formula: molecular formula; Compounds: names of substances; Class: classification of substances; Precursor (g/mol) : parent ion; Mass (g/mol) : molecular weight; RT (min) : retention time; Adduct: adduct mode score: material quality score; PubChem CID: Substance PubChem database number; CAS: the CAS number of a substance; Sample columns: the relative content of all substances in each sample. B0, B1, B2, and B3 respectively represent Bulang pickled tea fermented for 0, 1, 2, and 3 months.

Table S2. VIP, p(1), pcorr(1) value of 35 differential metabolites of Bulang pickled tea with different fermentation time

ID	Compounds	B0vsB1			B1vsB2			B2vsB3		
		VIP	p(1)	pcorr(1)	VIP	p(1)	pcorr(1)	VIP	p(1)	pcorr(1)
MW0126595	strictosamide	0.63	-0.04	-0.47	1.34	-0.13	-0.85	1.31	0.09	0.77
MW0106523	DL-o-tyrosine	1.34	0.21	1.00	1.30	-0.14	-0.81	1.25	0.14	0.72
MEDP1126	(R)-(-)-2-phenylglycine	1.34	0.25	1.00	1.28	-0.16	-0.80	1.27	0.17	0.73
MEDN0050	L-theanine	1.29	0.32	0.95	1.57	-0.40	-1.00	1.58	-0.24	-0.92
MW0009353	penoxsulam	1.34	0.17	1.00	1.05	-0.04	-0.66	0.29	0.01	0.21
MW0009295	oxamflatin	1.34	0.17	1.00	0.59	-0.08	-0.37	0.60	0.10	0.40
MW0053814	harpagoside	0.55	0.01	0.42	0.57	-0.04	-0.37	1.35	-0.13	-0.79
MW0130157	multinoside A	1.07	-0.05	-0.81	1.48	-0.10	-0.93	1.38	0.09	0.79
MW0007231	gallic acid	1.25	-0.17	-0.92	1.25	0.15	0.78	0.61	0.07	0.36
MW0102797	thromboxane	0.47	0.01	0.37	1.57	-0.19	-0.99	1.00	0.13	0.57
MW0055176	myricetin	0.49	-0.02	-0.34	1.50	0.19	0.95	1.24	-0.13	-0.73
MW0010635	occidentoside	0.40	0.01	0.32	1.57	-0.12	-0.99	1.71	0.16	1.00
MW0137913	delphinidin	1.14	0.07	0.84	1.58	-0.11	-1.00	1.68	-0.10	-0.99
MEDL01764	maritimetin	1.21	0.16	0.91	1.17	0.13	0.73	1.11	-0.13	-0.63
MW0108349	morin	0.98	0.10	0.75	1.44	0.17	0.91	1.11	-0.10	-0.63
MW0127753	(+)-gallocatechin	1.33	0.21	1.00	1.15	-0.22	-0.73	0.87	-0.17	-0.51
MW0124156	fonsecin	1.29	0.02	0.96	0.78	0.02	0.51	1.38	0.12	0.80
MW0124151	fluroxypyr-meptyl	1.07	0.10	0.81	0.45	0.04	0.30	0.34	0.01	0.25
MW0139178	N-trifluoroacetyladriamycinol	1.34	0.13	1.00	0.84	0.03	0.53	1.05	0.03	0.66
MW0137945	dichamanetin	1.34	0.10	1.00	1.05	-0.02	-0.66	0.29	0.01	0.21
MW0138905	malvidin	1.32	0.10	0.99	1.54	0.10	0.98	0.88	0.04	0.53
MEDP1060	8-hydroxyquinoline	1.33	0.11	0.99	1.42	-0.07	-0.89	1.01	0.04	0.58
MEDP0453	indole	1.31	0.13	0.97	1.41	-0.08	-0.89	0.84	0.04	0.47
MW0138810	longistylin C	0.40	0.01	0.32	0.72	-0.09	-0.45	1.02	-0.15	-0.61
MEDN0161	guanosine 3',5'-cyclic monophosphate	1.26	0.07	0.94	1.51	-0.10	-0.96	1.52	-0.09	-0.90
MW0015222	7-methylxanthine	1.32	0.08	0.99	1.25	-0.08	-0.80	1.50	-0.12	-0.89
MW0169913	theobromine	0.93	0.05	0.68	1.47	-0.12	-0.94	1.67	-0.22	-0.98
MW0126643	1,3,7,9-tetramethyluric acid	1.34	0.26	1.00	1.32	-0.06	-0.84	1.56	-0.09	-0.92
MADN0551	3,5-dihydroxy-4-methoxybenzoic acid	1.24	0.06	0.93	0.95	0.07	0.58	1.09	-0.10	-0.64
MW0116757	1-[(3,5-Dimethyl-1,2-oxazol-4-yl)sulfonyl]piperidine-4-carboxylic acid	0.26	-0.02	-0.21	0.74	0.05	0.48	1.63	-0.13	-0.93
MW0013751	3,3-dimethylacrylic acid	0.03	0.00	0.02	0.10	-0.01	-0.06	1.30	0.16	0.76
MW0114986	neochlorogenic acid	1.30	0.14	0.97	0.06	0.00	-0.02	0.71	0.04	0.39
MW0136221	6-{2-[6-carboxy-5-(2,4-dihydroxyphenyl)-3-methylcyclohex-2-en-1-yl]-3-hydroxy-5-[6-hydroxy-7-(3-methylbut-2-en-1-yl)-1-benzofuran-2-yl]phenoxy}-3,4,5-trihydroxyoxane-2-carboxylic acid	0.54	0.05	0.38	1.29	-0.19	-0.81	1.44	0.25	0.85
MW0115839	(2E)-3-(1H-indol-2-yl)-2-propenoic acid	1.32	0.22	0.98	1.30	-0.10	-0.81	1.14	0.09	0.68
MEDP0039	betaine	1.16	-0.07	-0.86	1.14	0.16	0.73	0.57	0.12	0.32

Notes: ID: indicates the substance number; Compounds: names of substances; B0, B1, B2, and B3 respectively represent the peak areas of Bulang pickled tea fermented for 0, 1, 2, and 3 months, VIP: variable importance in projection; p (1): covariance; pcorr (1): correlation.
